# Alumni satisfaction in terms of classroom infrastructure, lecturer professionalism, and curriculum

**DOI:** 10.1016/j.heliyon.2021.e06679

**Published:** 2021-05-27

**Authors:** Retnowati Wiranto, Slameto Slameto

**Affiliations:** President University, Jl. Ki Hajar Dewantara, Jababeka Cikarang Indonesia

**Keywords:** Alumni satisfaction, Professionalism, Curriculum quality

## Abstract

In an effort to improve the quality of higher education, it is necessary to find the main predictors or determinants of the quality requested. One of the indicators is alumni satisfaction. Therefore, the problems of this research are to determine the satisfaction of the Educational Management Program alumni and to examine the variables, facilities and infrastructure, professionalism of lecturers, and curriculum relevance, which determine the alumni satisfaction. This was an ex-post facto study. The research design used an ex-post facto method because this method aims to find causes that allow changes in behavior or phenomena, in this case alumni satisfaction with the teaching and learning process. The study data was obtained from the alumni of the Educational Management Program who currently work in one selected regency. A total of 36 alumni were involved as samples. The data collection instrument used by the researchers was in the form of a scale consisting of 27 items that had been proved reliable and valid. The results showed that most alumni had a high level of satisfaction, and there was 1 model that determined an alumni satisfaction of 36.10%, namely for the professionalism of lecturers.

## 1 Introduction

Education is a necessity for human life. Hence, as it is easier to gain access to education, achieving a quality education is prioritized today (Paul, 2019). In numerous countries, efforts to satisfy alumni have resulted in the emergence of various kinds of higher education institutions as alternative choices to traditional universities. The expansion of a strong employer-oriented non-university sector combined with the workforce needs of every region and locality is one of the recent major structural alterations for higher education systems (Grubb, 2003; OECD, 2005). Private universities are generally focused on the academic sphere, such as students' academic experiences, the students for every study program, high quality programs, and faculty credentials. To ensure their success and competitiveness, universities should know what is significant for students and try to meet their expectations (Humphreys, 2018).

College management is increasingly concerned about the education quality. It commonly has to deal with the difficulty of devising measurement indicators and recognizing the aspects of a quality education. One method to cope with such a matter is SERVQUAL, a system that uses the gap between perceived experience and customer expectations as a gage of service quality to check the quality of services provided by professionals. Hampton utilized this method to find out and test the importance of several components of education quality, such as students' perceptions and expectations which are frequently molded and affected by their cultural and environmental backgrounds. As a result, it is pertinent to direct the focus of future research toward achieving a high-quality education.

Alumni satisfaction is essential as it can maintain good university/alumni relations and increase the chance for future alumni to donate funds, which is even more necessary in this challenging economy (Cummins, 2011). A university should examine where satisfaction setbacks have happened and try to see if there is a specific cause (job market reduction) and what can be done to improve alumni satisfaction.

The Educational Management Master's Degree Program (EM-MP) of a private higher education institution in Central Java, Indonesia, applies a 2013 competency-based curriculum after implementing it 4 years ago. The needs and demands have shifted over time at local and global levels, such as the creation of the Asian Economic Community (AEC). An evaluation of the curriculum according to customer satisfaction, especially students, is essential in revising the curriculum to match with AEC. One of the aims of the Educational Management Program is to acquire high satisfaction in the academic community. If the academic community is pleased with the services received, they will probably return to make repeat “purchases” and even recommend EM-MP to their friends and family members.

In retrospect, while alumni are still active on campus, the finding of Gibson (2010) who researched and summarized the conclusions of the expanded research in the past 15 years regarding aspects of the most significant contributions of student satisfaction in terms of their academic experience revealed that the main determinants of student satisfaction were summarized using a set of common variable names and attributes. It was found that the attributes of the academic program itself were the most significant for overall student satisfaction. This finding is interesting because student satisfaction ultimately determines satisfaction later after becoming alumni. These attributes comprise the teaching quality and curriculum as well as the achievements of students' learning and their career aspirations. The availability and quality of the services and facilities (infrastructure), as in offering ICT guidance and support, are also considered significant, despite the mixed conclusions regarding the significance of the physical aspects of the services provided. Nevertheless, the way academic personnel and services respond is also essential. Moreover, studies on alumni satisfaction and their implications worldwide are displayed in the following table (see Table 1):

**Table 1:** Research on alumni satisfaction from various parts of the world.

No	Region/Country	Researcher (year)	Title	Relevant results	Implications
1	Asia: Malaysia	Thian (2014)	Institutional factors that contribute to educational quality at a private higher education institution in Malaysia.	A significance to the existing literature regarding the quality, quality assurance, and strategies to manage the quality of education in private tertiary institutions was created.	Malaysia and other developing countries gain the requirements for good application to meet the quality of education at the level of private tertiary institutions.
	Turki	Turan et al. (2015)	Explaining career satisfaction on the basis of alumni satisfaction, gap period, and gender.	In some fields of research, in particular the concept of career satisfaction has not been adequately evaluated.	The need for an educational model based on alumni literature that is able to explain the career satisfaction of their alumni.
2	Europe	García-Aracil (2009)	European graduates' level of satisfaction with higher education.	The social environment and curriculum of the study programs were aspects the graduates were satisfied with the most, but a lack of research involvement, and insufficient course materials caused dissatisfaction among the graduates.	Administrative aspects are required to boost students' satisfaction. In the same way, the improvement of undergraduate education quality should be carried out hand in hand with the improvement of the position as a research institution.
3	Africa/Kenya	Kara et al. (2016)	Educational service quality and student satisfaction in public universities in Kenya.	They discovered 10 dimensions of the quality of educational services in universities that can be relied upon. Lecturer: professionalism, quality of teaching methods, reliable examinations, and obtained learning advantages. Facilities and infrastructure: teaching media, Internet accessibility, learning modules accessibility, campus surrounding quality, and library services. Student services: quality of administrative services and quality of student welfare services.	The courses being offered by the university should guarantee the future demands of graduates. In accordance with the market needs, the curriculum must be reviewed periodically to meet the learning experiences of students.
4	Australia/Sydney	Martin et al. (2000)	Graduate satisfaction with the university and perceived employment preparation.	Preparing graduates to meet the workplace requirements is in fact difficult to actualize by the universities.	The need to identify what factors contribute to students' preparations for work and the need for graduate feedback (which is within the control of the university).
5	USA	Cabrera et al. (2003)	Alumni survey.	A study of more than 270 sources (books, articles, and institutional reports) about alumni research.	The discovery of three conceptual approaches to further alumni research based on: 1. outcomes, 2. alumni involvement and competence, and 3. alumni grants.

Based on the research presented above, it is known that feedback from alumni is important. However, there has been a scarcity of research on alumni satisfaction (Alves and Raposo, 2007), particularly that related to the factors available within the university control systems, namely to prepare students for the workforce after graduating from the institutions (Martin et al., 2000). According to an intensive Google Scholar tracking, research on increasing the quality of management in higher education institutions based on alumni satisfaction according to the students' engagement and competencies has not been found. Some studies reviewed during the tracking process were from Cabrera et al. (2003); and exceptions for the quality improvement of a higher education institution (e.g., the implementation of ISO management) in improving student or alumni satisfaction (Ariff et al., 2015). Furthermore, research on alumni satisfaction is fundamental to improve the understanding of the educational process and provide a good quality of education (Delaney, 2004).

The main purpose of this research is to explore strategies in improving the quality of educational management programs. Therefore, it is important to find the determinant factors within the university control that contribute to the students' preparation programs after graduating from the institutions (Martin et al., 2000). The determinant factors in this research are crucial and belong to factors that have absolute characteristics within the cause-and-effect relationships. They also become the causal factors available within the university or individuals themselves.

The factors/variables influencing the alumni satisfaction are related to: (1) providing quality educational experiences to students (Sun et al., 2007), and clarity of design, interactions with instructors, and active discussions among course participants (Swan, 2001); (2) ensuring that instructional effectiveness comprises fourteen items which assess students' academic experiences, including the curriculum (Elliott and Healy, 2001); and (3) providing good constructive advice related to the curriculum and the availability of classroom facilities and infrastructure (Kardoyo et al., 2018). The factors/variables that function as determinants which influence the alumni satisfaction are: (1) the lecturer's professionalism, (2) the relevance of the curriculum, and (3) the quality of classroom facilities and infrastructure.

From the above explanation, it is reasonable to predict both theoretically and empirically that learning facilities, professionalism or lecturer performance factors, and curriculum are predictors of the quality of educational services (academic) received by students which, in turn, determine students' satisfaction both during the study and after graduation. Alumni satisfaction assessment is an important element in providing better, more efficient, and more effective services through management. However, a problem regarding the predictors which become a major determinant has not yet been found.

The research problems were formulated as follows: 1) determine the satisfaction level of alumni of the Educational Management Study Program that was developed through the 2013 curriculum, and 2) decide the variables: a) the availability and quality of facilities (infrastructure) in lecturing services, b) the professionalism or performance of lecturers, and c) the curriculum, which determine the alumni satisfaction. Considering that the variety of education quality at the private higher education institution level has been a serious problem for developing countries (Thian, 2014), the findings of this study will be significant for the management of research based on quality improvements in higher education, which have often been overlooked in the quality management design of higher education.

## 2 Theoretical review

In order to achieve a high level of alumni satisfaction, higher education (HE) must pay attention to the quality of services provided. The quality of HE services is closely correlated with student/alumni satisfaction (Tuan, 2012). The quality of service is able to provide an impetus to students to establish strong relationships with HE. In the long-run, this association will encourage private universities to carefully understand students' expectations and needs. In this way, satisfaction can create loyalty.

Alumni satisfaction is one of the prominent indicators which determine the education quality in HE management. Therefore, this should be sought after constantly. Satisfaction is the feeling of an individual who is satisfied after comparing reality with the expectations he/she receives for a product or service (Kotler and Susanto, 2000). The factors of alumni satisfaction with higher education services include service quality, emotional factors, cost, performance, speed, convenience, comfort, friendliness, equipment completeness, reliability, conformity to specifications, endurance, service ability, aesthetics, product characteristics, service, location, facilities, image, atmosphere, and communication (Budiastuti, 2002).

Facilities and infrastructure are often called educational facilities and infrastructure. Educational facilities mean everything in the form of tools or media that can be used to achieve educational goals, while educational infrastructure is defined as everything that becomes the main support for the implementation of the educational process (Azis, 2017). The intended educational facilities and infrastructure include furniture, consumables, books and other learning resources, teaching aids and/or educational media, educational equipment, and other equipment needed to support an orderly and continuous learning process. Meanwhile, educational infrastructure includes administrative rooms, canteen rooms, electricity and services, installations, classrooms, leadership rooms, laboratory rooms, teaching rooms, library rooms, production unit rooms, workshop rooms, and other spaces/places; for instance, playgrounds, creative places, sports venues, land, and buildings.

One definition of the profession, as mentioned by Page and Thomas (Amir, 2010), is described as being very prestigious at work and can be called professional when it produces benefits for social services, which can be done with systematic knowledge gained from academic education and continuous training, which has a high autonomics and code of ethics. A profession is usually associated with a professional code of ethics, professional associations, certification processes, and special licenses for professions. A “professional” means someone who holds a position or job that is carried out with high skills carefully and according to the science and education one takes. “Professionalism” is the commitment of members of the profession to continuously improve their abilities. “Professionalization” is a process of time travel that makes a person or group of people become professionals. Professionalism of lecturers (Hidayati and Siswati, 2018) is expected to have high performance that can satisfy all stakeholders, especially students and parents as well as the wider community. Besides satisfying the stakeholders, high performance will also satisfy the individual, since professional spiritual satisfaction is the final compensation expected for a job. As professionals, lecturers are required to have a number of competencies to carry out their duties correctly and be called as professionals. These competencies consist of (1) the fields of study, (2) the understanding of students, (3) learning and education, and (4) personal competence and professional development.

Educational ideas in practice are the realization of the curriculum. The word ‘curriculum’ is etymologically derived from a Latin word whose meaning is for a track or racecourse. That is why curriculum is defined as a course or syllabus. Nowadays, its definition has expanded and covers all lesson plans from an educational academy. In the same way, in higher education, the curriculum is supposed to be a matter that is communicable to teaching personnel, open to any ideas, and practicable. Moreover, the curriculum consists of three steps, namely the plan, materials, and experiences which students obtain from the learning environment.

The curriculum needs to be ready for any shift in values and educational demands as long as they are advantageous. When it is further elaborated as being more than just a set of syllabi or plans, the curriculum requires more comprehensive content than merely learning the materials. Accordingly, the curriculum must at least consist of four aspects, including the content, teaching and learning methods, assessment, and evaluation. The relationship of these four aspects can be seen in curriculum maps. These maps present the curriculum's crucial characteristics thoroughly. The curriculum maps also feature the curriculum systematic organization in the form of a diagram, so that the curriculum can be input to a computer directory based on its organizational basis. Thus, what is being prepared, such as the planning, materials to deliver, and experiences to be given to students can be simply reviewed in terms of their relationship with the curriculum (Prideaux, 2003). This role implies the importance of a curriculum that it must be able to develop something new in accordance with developments and the needs of the community not only in the present but also in the future. The curriculum must support things that help students develop their potentials to gain new knowledge, new abilities, and new ways of thinking for their future lives. Variables that reflect the curriculum structure are important predictors of satisfaction with students' learning environments (Smith and Worsfold, 2014).

## 3 Method

This research was ex-post facto and conducted on the basis of the assessment of the alumni of the EM - MP of a private university in Central Java, Indonesia. The population of this study amounted to 156 alumni of the Educational Management Master Program of a private university in 5 regions, namely in the Temanggung, Grobogan, Kendal, Demak, and Semarang regencies. The study used an ex-post facto research design because this type of design aims to find causes that allow changes in behavior; symptoms or phenomena which are caused by an event; and symptoms, behavior, or phenomena that cause changes to the independent variable as a whole to occur. Ex-post facto research is an experimental method which also tests the independent variables that have occurred as a whole. An advantage of using the ex-post facto method is that it is in accordance with the circumstances that cannot be done by experimental research.

The research sample was chosen randomly from one of the regencies, namely Temanggung Regency. There were 36 alumni in Temanggung Regency. The number of the sample was in accordance with the requirement suggested by Cohen et al. (2007) who proposed that: “A sample size of thirty is held by many to be the minimum number of cases if researchers plan to use some form of statistical analysis on their data.” The participants had graduated from their schools in 3–6 years. All of them have been working as educators or educational staff, and 60% of them are females.

In accordance with the formulation of the problem, this study employed quantitative inferential research which investigated the inferential relationships between two or more variables that could explain the symptoms by analyzing the effects of variables X_1_ (availability and quality of facilities and infrastructure in lecturing services), X_2_ (professionalism or lecturer performance), and X_3_ (curriculum relevance) on Y (the level of satisfaction of the Educational Management Program alumni of a private university in Central Java). The next step was to find the determinants of the relevant three independent variables. Afterwards, the data collection was done in the second semester of 2017.

### 3.1 Educational facilities and infrastructure

It is appropriate for educational institutions to have adequate infrastructure, so that teaching and learning activities will be effective and satisfy students. Infrastructure provides the right environment and makes the teaching and learning interesting and comfortable (Paul, 2019). Educational facilities and infrastructure include access for disabled individuals and medical facilities, classroom and lecture theaters, computers and ICT multimedia, laboratories, libraries and sports facilities, and up-to-date modern and diverse courses.

### 3.2 Professionalism or lecturer performance

Starting from six main areas, Tichenor and Tichenor (2005) stated that effective teachers are categorized into two principal teachers as individuals and the remaining four categories are teacher responsibilities and practices. Finally, they are summarized into three statements, namely recognizing complexity, communicating clearly, and providing care with care.

### 3.3 Curriculum relevance

The four essential elements of the curriculum are content, teaching and learning strategies, the assessment process, and the objective achievement evaluation process. Hiim (2017) revealed that to ensure the relevance of the curriculum, authentic practical work is needed as a basis of the integration of subjects with practical work experience for students.

### 3.4 Alumni satisfaction

Turan et al. (2015) claimed that the satisfaction of alumni consists of eight statements, namely:

1. If I am accepted in a higher education (continued) institution, I will register to [university name].
2. I choose [name of university] as my college.
3. I gained adequate knowledge from my previous education to develop my hard skills.
4. The knowledge I gained from my previous education made my performance better than others from different universities in my first job.
5. The knowledge I gained from my previous education made me ready for my employment.
6. I choose the right university at [university name] to get an education.
7. I would recommend [the name of the university] to students who are interested in a career/business. I personally believe that I made a wise choice by the time I registered to [university name].

### 3.5 Instruments

This research belongs to quantitative research. The data for each variable constituted an ordinal scale expressed in terms of rank. The data for each variable studied was collected using a self-rating scale. According to Sugiyono (2002), a non-test instrument used to measure attitudes should fulfill the construct validity. Based on Sugiyono (2002), in order to calculate the internal reliability consistency, the Cornbach Alpha technique is used in the questions or statements in the research instruments. The alpha coefficient ranges from 0 to 1. When an item of measurement gains a value higher than 0.60, it is considered reliable. To measure the 4 variables of this study, a 32-item scale was developed with 4 alternative answers (score 1 = lowest, to score 4 = highest). At the time of the reliability testing, it turned out that 5 items were dropped because their item scores were less than 0.30. This analysis resulted in 4 factors to explain 79.197% of the respondents' data. The results of the factor analysis are presented in Table 2 below.

**Table 2:** Results of the factor analysis of the instrument validity and reliability.

Factor	Number & item number	Reliability of Cronbach's Alpha	Corrected Item -Total Correlation
1. Educational facilities	9 (1, 3, 10, 11, 12, 14, 16, 17, 18, 21, 26)	.896	.468 – .813
2. Alumni satisfaction	8 (7, 8, 13, 15, 20, 22, 27, 28)	.841	.380 – .708
3. Professionalism	5 (9, 29, 30, 31, 32)	.829	.453 – .774
4. Curriculum relevance	5 (4, 5, 19, 24, 25)	.768	.389 –. 678

Previously, the Campus Ethics Commission (Mr. Donald Samuel) stated that this instrument could be used.

### 3.6 Statistical hypothesis

Alumni satisfaction was measured using an ordinal scale consisting of 4 categories, namely very high, high, medium, and low. Among these 4 categories, there was one dominant category level. Among the 3 independent variables, namely X_1_ (availability and quality of facilities (facilities and infrastructure) in lecture services), X_2_ (professionalism or lecturer performance), and X_3_ (curriculum relevance), the researchers searched for the determinants (which had positive and significant effects) on Y (the level of satisfaction of the alumni of the Educational Management Program of a private university in Central Java, Indonesia).

### 3.7 Data analysis techniques

The researchers conducted a data analysis in two stages. In the first stage, each variable was analyzed using the frequency distribution and statistical indexes. In the second step, the data was analyzed using a multiple linear regression analysis. The best regression model is the one that is able to explain the dependent variable characteristics by choosing one from many independent variables within the data using the comparison criteria of the adjusted R^2^ and S^2^. Another model that is considered more suitable to obtain the best model is the stepwise method (Pujilestari, 2016). In this research, this method was used to determine the model. From this analysis, the researchers found a relationship model (causal model). Following this step, the researchers calculated the data using SPSS Version 24.

The research method design was in accordance with the applicable provisions on campus (“Research and Community Service Ethics Commission” of President University).

## 4 Research results

### 4.1 Descriptive analysis results

After the data was collected from 29 items of a self-rating scale, they were reduced to 4 variables. With the help of the SPSS program for Windows Version 24, the results of the descriptive analysis are presented in the following table.

In Table 3 above, the results of the descriptive analysis indicate that most of the alumni of EM – MP considered the variable of facilities and infrastructure services (X_1_) to be at a high level. They even believed that lecturer professionalism (X_2_) deserved to be at a very high level, while the relevance of the curriculum (X_3_) was at a high level. In addition, the level of satisfaction of the alumni of the Educational Management Program (Y) occupied a high level (Mean > Median).

**Table 3:** Descriptive analysis results.

Variable	Mean	Median	Sd.	Min.	Max.
X_1_ Educational facilities	3.4286	3.00	.51355	3.00	4.00
X_2_ Professionalism	3.8611	4.00	.35074	3.00	4.00
X_3_ Curriculum relevance	3.2857	3.00	.45835	3.00	4.00
Y Alumni satisfaction	3.2870	3.00	.60373	3.00	4.00

### 4.2 Hypothesis testing

To find out whether the three independent variables (X) affected the satisfaction of the Educational Management Program (Y), a hypothesis test analysis was performed. If it was proven true, then the determinant variable and its influence had to be found. The results of the hypothesis testing using a regression analysis are presented in the following two tables.

As described in Table 4 above, the regression test results in model 1 showed the value of R = 0.617 and Adjusted R^2^ = 0.361 or 36.10%. To ensure that the R^2^ obtained a significant result, the ANOVA analysis results are presented below.

**Table 4:** Model (summary) of the determinants’ influence on alumni satisfaction.

Model	R	R Square	Adjusted R Square	Std. Error of the Estimate
1	0.617^a^	0.381	0.361	1.95223

a Predictors: (Constant), X_2_.

In Table 5 above, the value of F in model 1 was 19.674, and at a significance level of 0.000. Since the significance level was less than 0.05, the professionalism of lecturers (X_2_) gained a positive and significant effect on the satisfaction of the alumni of the Educational Management Program (Y) of 36.10%. Meanwhile, facilities and infrastructure services (X_1_) and curriculum quality (X_3_) were excluded from this model, meaning that these two variables were not determinants of alumni satisfaction (Y). To find out the role of the predictor, the following table is presented.

**Table 5:** ANOVA analysis results of alumni satisfaction predictors.

Model		Sum of Squares	df	Mean Square	F	Sig.
1	Regression	74.983	1	74.983	19.674	0.000^a^
	Residual	121.959	32	3.811		
	Total	196.941	33			

Dependent Variable: Y.

a Predictors: (Constant), X_2_.

Based on Table 6, the coefficient of alumni satisfaction of the Educational Management Program achieved 1 predictor (model), namely model 1 or the lecturer professionalism variable (X_2_). In model 1, the value of T amounted to 4.436 with a significance of 0.000. This result is in line with the hypothesis that is H_1_: b_1_ ≠ 0 (there is a main predictor of alumni satisfaction levels). Furthermore, the data in model 1 shows that the lecturer professionalism variable (X_2_) (obtained R = 0.617 and Adjusted R2 = 0.361 with a significance level of 0.000) became the determinant of alumni satisfaction. This means that 36.10% of alumni satisfaction was determined by the level of lecturers' professionalism. Therefore, H_1_ which states the existence of a determinant/professionalism of lecturers (X_2_) can be accepted. On the other hand, the other variables, facilities and infrastructure services (X1) and curriculum quality (X_3_), were not determinants of the satisfaction of alumni of the Educational Management Program, because these were influenced by another variable that was not discussed in this research. For the ex-post research design, the researcher Cannot regulate the independent variable (Lammers and Badia, 2005).

**Table 6:** Coefficients.^a^

Model		Unstandardized Coefficient		Standardized Coefficients	T	Sig.
		B	Std. Error	Beta		
1	(Constant)	- 2.979	3.658		- .815	.421
	X_2_	4.193	.945	.617	4.436	.000

a Dependent Variable: Y.

## 5 Discussion

Indrawati (2011) in her research concludes that the Arithmetic Mental Education Institute in the city of Malang has not fully satisfied consumers, given the value of consumer expectations is still higher than the performance showed by the institution. Indrawati's findings are different from the findings of the researchers who found that most of the alumni gained a high level of satisfaction and even tended to be at a very high level. Quality service can certainly increase people's satisfaction and trust. Facilities and infrastructure are one of 3 variables that significantly contribute to student satisfaction (which ultimately determines the future satisfaction after becoming an alumni) with the education services provided (Kwan and Ng, 1999).

The findings of this study revealed that the level of alumni satisfaction was at a high level. Even so, there were still a few things that needed to be considered and used as input for future curriculum development, namely the competence of students in terms of the use of English as a means of communication, the use of ICT, and the field of research, especially related to scientific and professional development as a teacher or as a professional staff in business and industry fields. There are still a few problematic things experienced by graduates, particularly the ability to develop professionalism in the career field and the ability to adjust to changes or developments in science and technology (Sudirtha, 2013).

The results show that the determinant of alumni satisfaction was lecturer professionalism (model 1, amounting to 36.10%). Conversely, the role of quality of facilities and infrastructure services and curriculum relevance variable did not fulfill the requirements to be a predictor of satisfaction of alumni based on the data. In improving the quality of the Educational Management Study Program where alumni satisfaction is an important quality indicator, the findings of this study are very beneficial. The findings presented in model 1 indicate that the study program quality was measured not only until when students graduated from the university, but also until the alumni entered their respective work worlds. The model found that they were satisfied with the services of the study programs during college. This implies that the Educational Management Study Program should place quality in the satisfaction of its students, and further confirms that the quality of its graduates is in the hands of its own graduates (Suwartana, 2011). Thus, the quality management of study programs cannot be separated from the needs and expectations of graduates. The main keys are the professional quality of lecturers. On the other hand, the findings of the current study are not in line with the findings of Kwan and Ng (1999), who stated that educational facilities or infrastructure are one of 3 significant factors that contribute to student/alumni satisfaction with the educational services they receive.

The Educational Management Study Program has prepared its management to improve the service quality in order to equip its students with skills that are useful to make the graduates succeed and survive in the work world. These efforts were actualized by the stakeholders of the study program because success can be achieved due to satisfaction and the quality services (Abdullateef et al., 2011). Accordingly, an organization is encouraged to improve the management system based on clients' satisfaction, so that it can fulfill demands from the clients and deal with any criticism maturely. Once an organization is able to realize these concepts and serve its clients with accurate and punctual information, the organization will obtain performance values and multiple service quality in fulfilling customer satisfaction (SQM, 2007). Nowadays, client satisfaction is a key to a successful business in this intensely competitive era (Jamal and Naser, 2002). In addition, technology and infrastructure as parts of classroom facilities can also improve the rate of client satisfaction (Zhu et al., 2002), although this research did not support it.

To manifest the desired classroom lectures based on customer satisfaction, professional lecturers are needed. This refers to the efforts made as educators and learners of the realization of the role in higher education (Arikunto, 2012). The development of lecturer professionalism needs to be interpreted as a broad effort to improve competence, quality of learning, and the academic role of ICT-assisted lecturers. Lecturers are at the forefront of tertiary education. Therefore, they play a significant role in determining the quality of education and graduates. By referring to these theories, it is known that if lecturers have good quality, the tertiary institution will also have the same (Sy-Zain, n.d.).

Satisfactory results cannot be achieved merely by good programs and curriculum. Rather there should be high quality lecturers to amplify this situation (Sy-Zain, n.d.). It is because to run a good education program, an institution requires qualified or good lecturers. By having high quality lecturers, tertiary institutions will be able to formulate programs and curricula (based on ICT) to guarantee the birth of outstanding and high quality graduates (Sudiro, 2010).

Alumni are an integral part of the higher education institution when they provide valuable feedback on the service of the institution (Petratos and Calitz, 2019). Based on the findings of this study, alumni satisfaction (as a representation of higher education institution quality) is determined by the professionalism of lecturers and the quality of facilities and infrastructure services. Therefore, in order to increase the quality of management of higher education institutions, lecturers' professionalism needs to be improved (professional empowerment) by increasing the facility services and infrastructure innovatively. Janssen (2000) elaborated on the innovative working attitudes in the professional field by creating, introducing, and applying new ideas required in the performance of a group or organization based on the contributions and performance of the group or organization.

## 6 Conclusion

The present research offers important data and an analysis of the quality indicators to improve the quality of higher education (Educational Management Program) through an alumni survey regarding the classroom infrastructure, lecturer professionalism, and curriculum. The results of the research revealed that the level of satisfaction of most alumni is at a high level. One determinant is lecturer professionalism with a magnitude of influence of 36.10% (model 1). The role of the quality of classroom lectures, facilities, and infrastructure services, and the curriculum relevance variable are not a predictor of satisfaction of alumni based on the data.

These findings confirm the need for proper management of study programs of private universities based on alumni satisfaction with the support of lecturer professionalism quality. Lecturers who maintain and improve the customer satisfaction and have the need for professional empowerment need to be supported to increase the quality of management in a private higher education institution. The essence of professional empowerment is to enable lecturers to release and utilize their experiences, initiatives, knowledge, and wisdom. The process involves actions and programs which build lecturers' capacities to improve their own proficiencies and outcomes as well as the efficiency and effectiveness of their university. Such programs include training of trainers through internal workshops, tutorials, case studies, seminars, and apprenticeships.

## Declarations

### Author contribution statement

R. Wiranto, S. Slamento: Conceived and designed the experiments; Performed the experiments; Analyzed and interpreted the data; Contributed reagents, materials, analysis tools or data; Wrote the paper.

### Funding statement

This work was supported by President University (1500).

### Data availability statement

The authors do not have permission to share data.

### Declaration of interests statement

The authors declare no conflict of interest.

### Additional information

No additional information is available for this paper.
